# Editorial: Plant circadian rhythms

**DOI:** 10.3389/fpls.2022.1109900

**Published:** 2022-12-13

**Authors:** Xiaodong Xu

**Affiliations:** State Key Laboratory of Crop Stress Adaptation and Improvement, School of Life Sciences, Henan University, Kaifeng, China

**Keywords:** circadian clock, circadian rhythm, clock outputs, metabolic signaling, phytochrome, ABA

The circadian clock, a time-keeping mechanism, anticipates the cyclic changes of environmental signals (e.g., light, temperature, humidity) and confers environmental fitness to organisms (Harmer et al., 2022). Multiple nuclear-localized core oscillators sequentially expressed from dawn to night comprise the interlocked transcriptional-translational feedback loops (TTFLs), which drive and fine-tune the endogenous 24-h rhythms, assigning the temporal signature of period length, phase, and amplitude to physiological and metabolic processes (Nohales and Kay, 2016; Xu et al., 2022b). The expression of circadian core oscillators is sensitive to external timing factors, such as discontinuous light or temperature pulses throughout the day that alter the phase of circadian rhythm (Salomé and McClung, 2005). Transduction components of light and temperature signaling pathways are involved in the entrainment or resetting of the circadian clock by external cues. Functional circadian clock is critical for plant adaptation to photoperiodic flowering, nodulation, seasonal dormancy, senescence, and for crop hybrid vigor, stress resistance, and natural selection and domestication of ancestral wild species (Seo and Mas, 2015; Mora-Garcia et al., 2017; Xu et al., 2022a). The study of the circadian clock system has focused on molecular architecture based on TTFLs, timing factors (*Zeitgebers*), and clock output pathways.

The core oscillators contribute to the parameter setting of circadian rhythms, including clock pace (Somers et al., 1998; Nohales and Kay, 2016). In recent years, new components have been identified that are involved in TTFLs together with core oscillators, such as LIGHT-REGULATED WD1, LWD2, NIGHT LIGHT-INDUCIBLE AND CLOCK-REGULATED/LNK1, LNK2, B-box (BBX)-containing proteins/BBX18, and BBX19 (Rugnone et al., 2013; Xie et al., 2014; Wu et al., 2016; Yuan et al., 2021). In addition, cell-autonomous clocks ensure the independence of the rhythmicity in individual organs and tissues, probably due to differences in physiological functions and environmental cues they are exposed to. To accomplish a certain physiological process for the whole plant, circadian oscillators communicate with each other in a hierarchical network, which is similar to the synchronization between the suprachiasmatic nucleus (SCN) central clocks and peripheral clocks in mammals (Endo et al., 2014; Takahashi et al., 2015). The shoot apex potentially plays a more dominant role than the root. Also, clock-regulated photosynthetic product sucrose oscillates in the leaves and acts as a metabolic signal to feedback entrain the circadian clock. It is known that *PSEUDO-RESPONSE REGULATOR7*/*PRR7* and basic-leucine zipper (bZIP) transcription factor gene/*bZIP63* orchestrate clock pace and circadian phases in the sucrose response (Haydon et al., 2013; Frank et al., 2018).

In a recent Research Topic on plant circadian rhythms, Nimmo and Laird reported that *prr7-11* mutant exhibits a shortened period of *CIRCADIAN CLOCK-ASSOCIATED 1*/*CCA1:LUC* rhythm in both shoots and roots relative to wild-type plants, with wild-type plants displaying longer period in roots than in shoots. From scatter plotting of the data, the change in period length in *prr7-11* shoots was greater than that in roots, indicating that *PRR7* functions or contributes more in shoots than it does in roots. The authors found that *prr7-11* shoots were sensitive to light intensity. When exposed to 50 μmol m^-2^ s^-1^, *CCA1:LUC* rhythms in *prr7-11* decreased in amplitude after two days in constant light (LL96-144), compared to light/dark cycle (LD0-48) and the first two days in constant light (LL48-72), and gradually lost rhythmicity at 25 and 5 μmol m^-2^ s^-1^ light intensity. Contrary to shoots, the roots of *prr7-11* showed a decrease in circadian amplitude under free-running conditions, but maintained circadian rhythmicity under dim light. The contribution of *PRR7* to *CCA1:LUC* activity in shoot and root organs under various photoperiodic conditions can also be observed from this study. When switching between light and dark, the *CCA1* promoter activity in wild-type shoots and roots and in *prr7-11* shoots was sensitive to light cue, whereas the light response of *CCA1* promoter in *prr7-11* roots was not significant. Taken together, this study established the role of *PRR7* in regulating circadian rhythm and *CCA1* promoter activity in shoots and roots in light/dark cycle and constant light conditions.

The circadian clock acts on its timing signaling pathway. In light input of circadian system, the expression of light-regulated components exhibits robust 24-h rhythms that regulate the core oscillators and their target gene expression at specific times of the day (Wu et al., 2016; Liu et al., 2020). In this topic of plant circadian rhythms, Liu et al. found that the transcripts and proteins of *FAR-RED ELONGATED HYPOCOTYL1*/*FHY1*, which mediates the far-red light response, show oscillations in diurnal enrichment, peaking at ZT8 during daytime. qPCR results showed that transcript levels of *FHY1* were increased in the *cca1-1* mutant but were repressed in response to *TIMING OF CAB EXPRESSION1*/*TOC1* overexpression. The transcription of *FHY1* activated by FHY3 detected in yeast and tobacco cells was inhibited by the addition of *CCA1* and *TOC1*. Further, phyA protein was induced by far-red light in wild-type plant showing significant nuclear localization, which was suppressed by *CCA1* and *TOC1* overexpression. Thus, in the working hypothesis of far-red light signaling proposed by Liu et al, CCA1 inhibits *FHY1* expression in the early morning. As CCA1 expression decreases during the day, the inhibitory effect of CCA1 on *FHY1* is relieved around noon, causing *FHY1* transcript accumulation, which promotes phyA transport to the nucleus and activates far-red light signaling. In the evening, TOC1 represses *FHY1* again and shuts down phyA signaling.

Circadian core oscillators are mostly nuclear-localized transcription factors whose target genes mediate numerous plant pathways in response to environmental light, temperature, stress and metabolic signals (Harmer et al., 2000). Recently, Blair et al. investigated the involvement of circadian clock-regulated CYCLING DOF FACTOR6 (CDF6) in cold responses and flowering time. The transcript accumulation of *CDF6* showed a nearly 24-h rhythm under continuous light at 22°C (free-running conditions), with the peak occurring around dawn, indicating that *CDF6* expression is regulated by the circadian clock. In *CCA1*-overexpressing plants, the robustness of the *CDF6* rhythmic expression was reduced, i.e. the amplitude was down-regulated, suggesting that CCA1 potentially negatively regulates *CDF6* transcription. The CDF6 protein is localized in vasculature. A low temperature of 10°C for 1 h significantly induced *CDF6* expression, but the peak time was not different from 22°C. However, when *CCA1* was highly expressed in vasculature in a *SUC2:CCA1* transgenic line, the *CDF6* peak expression appeared to be shifted forward by about 4 h. This result suggests that the circadian clock gates *CDF6* rhythmic expression in response to cold stress. In addition, genome-wide gene expression analysis by RNA-sequencing in wild-type, *cdf6* mutant and *SUC2:CDF6* identified that the differentially expressed genes (DEGs) resulting from the cold treatment included key components of the circadian clock, clock-mediated photoperiodic flowering, and the vernalization pathway. Analysis of circadian rhythms of the *CO* and *FT* transcripts of the photoperiodic flowering pathway showed that *CDF6* expression in vasculature altered the circadian phase and slightly inhibited the amplitude of the *CO* transcript level oscillation, resulting in the suppression of *FT* and the late flowering of *SUC2:CDF6* plants.

Circadian clock gating of ABA biosynthesis causes daily fluctuations of ABA levels, thereby regulating ABA-mediated plant growth and stress tolerance processes (Tallman, 2004; Atamian and Harmer, 2016; Belbin and Dodd, 2018). The *prr5-1*, a mutation in a core oscillator gene PRR5, led to an increase in stomatal opening but the mutant closed more readily when treated with ABA, suggesting that ABA can act in turn on the circadian clock (Jurca et al., 2022). In the proteolytic turnover of circadian clock proteins, ZTL acts as an F-box E3 ubiquitin ligase that recognizes phosphorylated PRR5 and TOC1 and drives their degradation (Fujiwara et al., 2008). Recently, Yu et al. found that *ZTL* overexpression and mutation caused defects in plant responses to ABA, including stomatal opening, root elongation, survival rate and water loss under drought stress. ZTL interacted with Mg-chelatase H subunit and putative ABA receptor, CHLH/ABAR, and caused CHLH polyubiquitination. *CHLH* transcripts and CHLH proteins appear to be more abundant in the early morning and less abundant at night, i.e., exhibiting diel oscillations. ABA treatment promoted ZTL phosphorylation and accelerated CHLH degradation through the proteasome pathway. The authors also generated the genetic material, *ztl-3 cch* double mutant, to confirm that *CHLH* is involved in regulating *ztl-3* hypersensitivity to ABA in stomatal opening and drought tolerance. In summary, this study proposes a working hypothesis that ABA signaling is involved in the post-transcriptional regulation of circadian clock proteins.

The synchronization of the circadian clock with the environmental cues allows for the rational distribution of photosynthetic products and nitrogen assimilation products in order to maintain tissue and organ homeostasis (Steed et al., 2021; Xu et al., 2022b). The circadian oscillators directly regulate the target genes involved in mitochondria and chloroplast energy metabolism. Cervela-Cardona et al. outline cellular energy generation and utilization in photosynthesis during the day and respiration during the night. Using the well-known TCA cycle enzyme fumarase in eukaryotes mitochondrial matrix, the authors illustrate how the circadian clock ensures ATP energy homeostasis in a 24 h day-night cycle. In addition, while reviewing the molecular architecture of circadian core oscillators, Venkat and Muneer elaborated the critical roles of clock-regulated photosynthesis, carbon assimilation and sugar metabolism in plant growth and senescence, and summarized the contribution of circadian clock-gated hormone signaling in plant defense responses.

In conclusion, knowledge of the circadian clock system facilitates the study of agronomic traits in crops, including the application of chronoculture (Steed et al., 2021). Each clock pace of physiological and metabolic rhythms determines crop photosynthetic efficiency, energy use efficiency, and stress resistance, etc. Evaluation of crop varieties for circadian parameters and modification of core oscillators will accelerate the development of high yielding and value-added crop germplasm and varieties.

## Author contributions

The author confirms being the sole contributor of this work and has approved it for publication.
